# Exploring neutrophil functionality in breast cancer progression: A review

**DOI:** 10.1097/MD.0000000000037654

**Published:** 2024-03-29

**Authors:** Emmanuel Ifeanyi Obeagu, Getrude Uzoma Obeagu

**Affiliations:** a Department of Medical Laboratory Science, Kampala International University, Kampala, Uganda; b School of Nursing Science, Kampala International University, Kampala, Uganda.

**Keywords:** breast cancer, cancer immunology, cytokines, immune cells, inflammation, neutrophils

## Abstract

Breast cancer remains a pressing global health concern, with a myriad of intricate factors contributing to its development, progression, and heterogeneity. Among these multifaceted elements, the role of immune cells within the tumor microenvironment is gaining increasing attention. In this context, neutrophils, traditionally regarded as the first responders to infections, are emerging as noteworthy participants in the complex landscape of breast cancer. This paper seeks to unravel the intricate and multifaceted role of neutrophils in breast cancer. Neutrophils, classically known for their phagocytic and pro-inflammatory functions, are now recognized for their involvement in promoting or restraining tumor growth. While their presence within the tumor microenvironment may exert antitumor effects through immune surveillance and cytotoxic activities, these innate immune cells can also facilitate tumor progression by fostering an immunosuppressive milieu, promoting angiogenesis, and aiding metastatic dissemination. The intricacies of neutrophil-tumor cell interactions, signaling pathways, and mechanisms governing their recruitment to the tumor site are explored in detail. Challenges and gaps in current knowledge are acknowledged, and future directions for research are outlined. This review underscores the dynamic and context-dependent role of neutrophils in breast cancer and emphasizes the significance of unraveling their multifaceted contributions. As we delve into the complexities of the immune landscape in breast cancer, a deeper understanding of the warriors within, the neutrophils, presents exciting prospects for the development of novel therapeutic strategies and a more comprehensive approach to breast cancer management.

## 1. Introduction

Breast cancer, one of the most prevalent malignancies affecting women worldwide, continues to be a formidable challenge for both patients and healthcare providers.^[1]^ The complexity of this disease lies not only in its varied molecular subtypes but also in the intricate interplay of cellular and molecular components within the tumor microenvironment.^[2]^ In recent years, the influence of immune cells on the development, progression, and treatment of breast cancer has come to the forefront of research and clinical interest.^[3]^

While the adaptive immune system, particularly T lymphocytes, has received substantial attention for its role in immunosurveillance and immune response against cancer, the innate immune system has been an area of increasing exploration.^[4]^ Among the innate immune cells infiltrating the tumor microenvironment, neutrophils, the body’s swift and early responders to infections, have garnered attention as influential participants in the breast cancer narrative.^[5]^

Historically perceived as acute inflammatory mediators, neutrophils have been the focus of extensive research within the context of infection and inflammation.^[6]^ However, their role in the neoplastic transformation of breast tissue, a distinct and multifaceted process, has remained comparatively enigmatic.^[7]^ This paper endeavors to shed light on the intricate and multifaceted role of neutrophils in breast cancer, recognizing them as key players within the tumor microenvironment.

Neutrophils are unique among immune cells for their potent antimicrobial activities, notably phagocytosis and the release of cytotoxic granules. Their versatility extends to participation in immune responses and modulation of inflammation.^[8–10]^ In recent years, growing evidence has suggested that these immune warriors may have both pro-tumorigenic and anti-tumorigenic effects within the dynamic environment of breast cancer.^[11–13]^

The objective of this review is to comprehensively explore the dualistic role of neutrophils within the context of breast cancer. We delve into the mechanisms underlying their participation, from immune surveillance and cytotoxic activities that hinder tumor progression to their contribution to immunosuppressive microenvironments that facilitate tumor growth. We examine the signaling pathways and molecular mechanisms governing neutrophil-tumor cell interactions, as well as their recruitment to the tumor site.

Moreover, we consider the clinical implications of neutrophils in breast cancer, including their potential as prognostic markers, therapeutic targets, and predictors of therapeutic

## 2. Breast cancer pathogenesis and immune microenvironment

In the context of breast cancer, understanding the pathogenesis and the immune microenvironment is pivotal for deciphering the intricacies of this complex disease.^[14]^ Breast cancer pathogenesis involves the series of events leading to the initiation, growth, and potential metastasis of cancerous cells within the breast tissue.^[1]^ Simultaneously, the immune microenvironment plays a central role in shaping the course of breast cancer, affecting tumor progression and response to therapy.^[15]^

Breast cancer arises from genetic and epigenetic alterations in the cells of the breast tissue, which can lead to uncontrolled growth and division.^[16]^ Genetic mutations or epigenetic changes occur in normal breast cells, leading to the transformation of these cells into cancerous ones.^[17]^ This phase often remains asymptomatic. The cancer cells begin to divide rapidly and form a tumor within the breast.^[18]^ The tumor may remain localized or invade nearby tissues. If the cancer cells gain the ability to invade surrounding tissues and enter the bloodstream or lymphatic system, they can travel to distant organs and form metastatic tumors. Breast cancer is not a single disease but comprises various subtypes, each with distinct molecular characteristics, hormone receptor status, and HER2/neu expression. These subtypes influence the disease’s behavior and response to treatment.^[19]^

The immune microenvironment within the breast tumor is a complex network of immune cells, stromal cells, and signaling molecules that influence tumor growth and immune responses.^[20]^ Tumor-infiltrating lymphocytes are immune cells, such as T cells and B cells, that infiltrate the tumor and can have both anti-tumor and pro-tumor effects.^[21]^ Myeloid-derived suppressor cells have immunosuppressive functions and can hinder the immune system’s ability to attack cancer cells.^[22]^ Tumor-associated macrophages can have varying roles, depending on their polarization.^[23]^ M1-polarized macrophages have anti-tumor effects, while M2-polarized macrophages can promote tumor growth.^[24]^ Cytokines and Chemokines are secreted by both immune and tumor cells and play a crucial role in regulating immune responses and shaping the tumor microenvironment.^[25]^

Understanding the immune microenvironment’s role in breast cancer pathogenesis is essential for tailoring therapeutic strategies.^[26]^ It can influence treatment decisions, such as the use of immunotherapies or targeted therapies that modulate the immune response. Furthermore, characterizing the immune microenvironment can aid in predicting patient outcomes and developing personalized treatment plans. The pathogenesis of breast cancer is a multi-step process involving genetic and epigenetic alterations, while the immune microenvironment within the tumor plays a pivotal role in influencing disease progression and treatment response. This interplay between pathogenesis and the immune microenvironment is central to the complex nature of breast cancer and its management.^[27]^

## 3. Neutrophil biology and function

Neutrophils are a type of white blood cell, or leukocyte, and are an integral part of the body’s innate immune system.^[28]^ They play a crucial role in defending the body against bacterial and fungal infections. Neutrophils are the most abundant type of white blood cell in the bloodstream and are characterized by their multilobed nuclei and granular cytoplasm.^[29]^ Neutrophils are produced in the bone marrow through a process called granulopoiesis.^[30]^ They originate from hematopoietic stem cells, which differentiate into myeloblasts, promyelocytes, myelocytes, metamyelocytes, and eventually mature neutrophils.^[31]^ Neutrophils go through several stages of development, each marked by specific changes in their morphology and cellular contents.^[32]^ Mature neutrophils are then released into the bloodstream. Neutrophils have a relatively short lifespan, typically ranging from a few hours to a few days. This short lifespan is partly due to their highly active and destructive role in the immune response.^[33]^

Neutrophils are phagocytic cells, meaning they can engulf and digest microorganisms, such as bacteria and fungi. They use pseudopodia (temporary extensions of their cell membrane) to envelop pathogens and form phagosomes.^[34]^ These phagosomes then fuse with lysosomes, which contain enzymes that break down the pathogens. Neutrophils can produce reactive oxygen species during phagocytosis. This oxidative burst helps to kill engulfed microorganisms by damaging their cell membranes and DNA.^[35]^ Neutrophils contain granules filled with enzymes and antimicrobial proteins. These granules can be released to extracellular spaces to combat infections. There are 3 types of granules: azurophilic (primary), specific (secondary), and gelatinase (tertiary) granules.^[36]^ Neutrophils can sense chemical signals produced by damaged tissues and invading pathogens. They migrate toward the source of these signals in a process called chemotaxis.^[37]^ Neutrophils can release extracellular traps composed of DNA, histones, and granule proteins. These traps, called neutrophil extracellular traps (NETs), capture and kill bacteria and other pathogens.^[38]^ Neutrophils can produce cytokines and chemokines that influence other immune cells, regulating the overall immune response.^[39]^ Neutrophils are involved in the resolution phase of inflammation, where they are removed from the site of inflammation by macrophages and other phagocytes.^[40]^ Neutrophils are highly effective against bacterial and fungal infections.^[41]^ They are less effective against viral infections.^[42]^

Neutrophils are essential for the body’s defense against infections, particularly those caused by bacteria and fungi.^[41]^ Their rapid response and ability to kill pathogens are critical in preventing infections from spreading. Neutrophils are a vital component of the innate immune system, working alongside other immune cells to protect the body from a wide range of infectious agents.^[43]^

## 4. Neutrophils in breast cancer

The role of neutrophils in breast cancer is an area of growing interest and research.^[44]^ Neutrophils, which are primarily known for their role in the innate immune system’s response to infections, have been found to have complex and context-dependent interactions within the tumor microenvironment of breast cancer.^[45]^ Neutrophils can have both pro-tumorigenic and anti-tumorigenic effects in breast cancer.^[46]^ Conversely, neutrophils can exhibit anti-tumor activities by engaging in cytotoxic actions, facilitating the immune response, and limiting tumor growth.^[47]^

Neutrophils that infiltrate the tumor microenvironment are often referred to as tumor-associated neutrophils (TANs).^[48]^ The phenotypic characteristics and functions of TANs can vary depending on factors such as the specific breast cancer subtype and the stage of the disease.^[19]^ Some TANs may exhibit an N1 phenotype with anti-tumor properties, while others may display an N2 phenotype with pro-tumorigenic characteristics.^[49]^ Neutrophils can release structures known as NETs.^[50]^ These web-like structures are composed of DNA, histones, and granule proteins. NETs have been implicated in promoting tumor progression, angiogenesis, and the formation of metastases in breast cancer.^[51]^ Neutrophils within the tumor microenvironment can influence the overall immune response to breast cancer.^[52]^ Their interactions with other immune cells, such as T cells and dendritic cells, can shape the immune landscape of the tumor.^[53]^ The presence and characteristics of TANs in breast cancer tissues have been associated with patient prognosis and response to treatment.^[54]^ High levels of neutrophil infiltration in breast tumors may be associated with poorer outcomes in some cases.^[55]^

Ongoing research is exploring the potential of targeting neutrophils or modulating their functions as part of breast cancer treatment strategies.^[56]^ Strategies aimed at altering the balance of pro-tumorigenic and anti-tumorigenic neutrophil functions are under investigation. The role of neutrophils in breast cancer is context-dependent and varies across different breast cancer subtypes and stages.^[57]^ Their impact on tumor progression may also depend on the specific molecular and genetic characteristics of the tumor.^[58]^

The involvement of neutrophils in breast cancer is a multifaceted and evolving field of study.^[59]^ While they have the potential to exert both pro-tumorigenic and anti-tumorigenic effects, their precise role depends on the specific tumor microenvironment and the immune landscape. Further research is needed to better understand the complex interactions between neutrophils and breast cancer and to explore potential therapeutic interventions that target these immune cells.

## 5. Mechanisms and signaling pathways of neutrophils in breast cancer

The mechanisms and signaling pathways involving neutrophils in breast cancer are complex and multifaceted.^[60]^ Neutrophils can have both pro-tumorigenic and anti-tumorigenic effects depending on various factors, including the specific breast cancer subtype and the stage of the disease.^[61]^ Neutrophils are recruited to the tumor site through chemotactic signals.^[62]^ Tumor and stromal cells release chemokines (such as CXCL1, CXCL2, and CXCL8) that attract neutrophils to the tumor microenvironment.^[63]^ This recruitment is mediated through chemokine receptors on the surface of neutrophils, particularly CXCR1 and CXCR2. Neutrophils can release pro-inflammatory cytokines, such as tumor necrosis factor-alpha (TNF-α) and interleukin-1 beta (IL-1β), in the tumor microenvironment.^[64]^ These cytokines can promote inflammation, tissue remodeling, and cell proliferation, contributing to tumor growth and progression.^[65]^

Neutrophils can release factors like vascular endothelial growth factor and matrix metalloproteinases that promote angiogenesis, the formation of new blood vessels within the tumor.^[66]^ Angiogenesis facilitates the delivery of oxygen and nutrients to the tumor, supporting its growth.^[67]^ Neutrophils can exert immunosuppressive effects by inhibiting the activity of other immune cells, such as T cells and natural killer cells, within the tumor microenvironment.^[68]^ This immunosuppression can hinder the body’s ability to mount an effective anti-tumor immune response. Neutrophils can release NETs, which are composed of DNA, histones, and antimicrobial proteins.^[69]^ These structures can trap and kill pathogens but have also been implicated in promoting cancer progression. NETs can contribute to tumor cell adhesion, migration, and angiogenesis.^[70]^

Neutrophils can directly interact with tumor cells through cell-to-cell contacts.^[71]^ These interactions can influence tumor cell behavior and signaling. Neutrophils may engage in cross-talk with tumor cells, affecting their survival, proliferation, and metastatic potential.^[72]^ Neutrophils can adopt an M2-like polarization state within the tumor microenvironment, similar to the M2 phenotype of macrophages.^[73]^ M2-like TANs may exhibit pro-tumorigenic properties, such as promoting angiogenesis and immunosuppression. Neutrophils can recruit regulatory T cells to the tumor microenvironment, which can suppress anti-tumor immune responses.^[74]^ This recruitment further contributes to immune evasion by the tumor.

It is important to note that the precise mechanisms and signaling pathways of neutrophils in breast cancer can vary depending on the specific subtype of breast cancer and the stage of the disease.^[75]^ Furthermore, the balance between pro-tumorigenic and anti-tumorigenic effects of neutrophils may depend on the tumor microenvironment and its specific characteristics. Research into these pathways continues to provide insights into potential therapeutic targets and strategies to modulate neutrophil functions in breast cancer treatment.

## 6. Clinical implications of neutrophils in breast cancer

The clinical implications of neutrophils in breast cancer are multifaceted and continue to be an area of active research.^[76]^ Neutrophils can have both pro-tumorigenic and anti-tumorigenic effects in breast cancer, and their presence within the tumor microenvironment can influence patient prognosis, treatment response, and clinical decision-making.^[77]^ The presence and characteristics of tumor-infiltrating neutrophils or TANs within breast cancer tissues have been associated with patient prognosis.^[78]^ High levels of neutrophil infiltration may be correlated with poorer outcomes in certain breast cancer subtypes, as they can contribute to an immunosuppressive and pro-tumorigenic microenvironment.^[79]^

Neutrophil presence and activity can serve as predictive biomarkers for therapeutic responses in breast cancer.^[80]^ The balance between pro-tumorigenic and anti-tumorigenic neutrophil functions may influence how patients respond to specific treatments, such as chemotherapy, immunotherapy, or targeted therapy.^[81]^ Emerging immunotherapies, such as immune checkpoint inhibitors, are being explored for breast cancer treatment.^[82]^ Neutrophils within the tumor microenvironment may be potential targets for these therapies to enhance anti-tumor immune responses.^[83]^ Strategies aimed at modulating the functions of neutrophils or altering the balance between pro-tumorigenic and anti-tumorigenic activities are under investigation. Potential approaches include targeting neutrophil chemotaxis, blocking neutrophil-derived factors, or promoting anti-tumor activities of neutrophils. Neutrophil profiling and assessment of their interactions with other immune cells may aid in risk stratification for breast cancer patients.^[84]^ Identifying patients with a high neutrophil-to-lymphocyte ratio or other neutrophil-related factors may help predict disease aggressiveness and inform treatment decisions.^[85–88]^

## 7. Potential for combination therapies

Combining therapies that target neutrophils with other breast cancer treatments, such as chemotherapy or targeted therapies, may improve therapeutic outcomes.^[89]^ Combinatorial approaches could address the dynamic and complex nature of neutrophil involvement in breast cancer.^[90]^ The clinical implications of neutrophils in breast cancer underscore the importance of considering the immune microenvironment in the context of patient care.^[91]^ Understanding the balance of pro-tumorigenic and anti-tumorigenic neutrophil activities is crucial for optimizing treatment strategies and improving patient outcomes. Ongoing research is expected to provide further insights into the role of neutrophils in breast cancer and their potential as therapeutic targets.

## 8. The role of neutrophils on different breast cancer subtypes (ER+, HER2+, and TNBCs)

Neutrophils, a type of white blood cell, play diverse roles in the tumor microenvironment and can impact breast cancer progression and treatment response across different subtypes, namely ER+ (estrogen receptor-positive), HER2+ (human epidermal growth factor receptor 2-positive), and TNBC (triple-negative breast cancer).

### 8.1. Neutrophils in ER+ breast cancer

Studies have shown that higher levels of neutrophils in the tumor microenvironment of ER+ breast cancer can be associated with poorer outcomes, including decreased overall survival and increased risk of metastasis.^[26,92]^ Neutrophils might promote tumor growth and progression through the release of factors that enhance angiogenesis (formation of new blood vessels) and the suppression of immune responses against the tumor. Neutrophils can also contribute to treatment resistance by creating an immunosuppressive environment, reducing the effectiveness of certain therapies like hormone therapy.

### 8.2. Neutrophils in HER2+ breast cancer

Neutrophils have been observed to infiltrate HER2+ tumors and contribute to the development of resistance to HER2-targeted therapies, such as trastuzumab.^[93,94]^ They may facilitate resistance through the secretion of factors that promote tumor growth and suppress the immune response against the cancer cells. Neutrophils can also interact with other immune cells and cytokines within the tumor microenvironment, affecting the response to targeted therapies.

### 8.3. Neutrophils in TNBC

In TNBC, the role of neutrophils appears to be more complex and context-dependent. Neutrophils can exhibit both tumor-promoting and tumor-inhibiting functions in TNBC.^[95,96]^ They may contribute to the suppression of tumor growth by enhancing immune responses and exerting cytotoxic effects on cancer cells. However, in certain circumstances, neutrophils can also facilitate tumor progression by promoting inflammation, angiogenesis, and immunosuppression.

Overall, the role of neutrophils in breast cancer subtypes is multifaceted and can vary depending on the specific tumor microenvironment, the interplay with other immune cells, and the stage of the disease. Understanding these complexities is crucial for developing targeted therapies that leverage the immune system to combat breast cancer effectively across its diverse subtypes.

**Figure 1.**
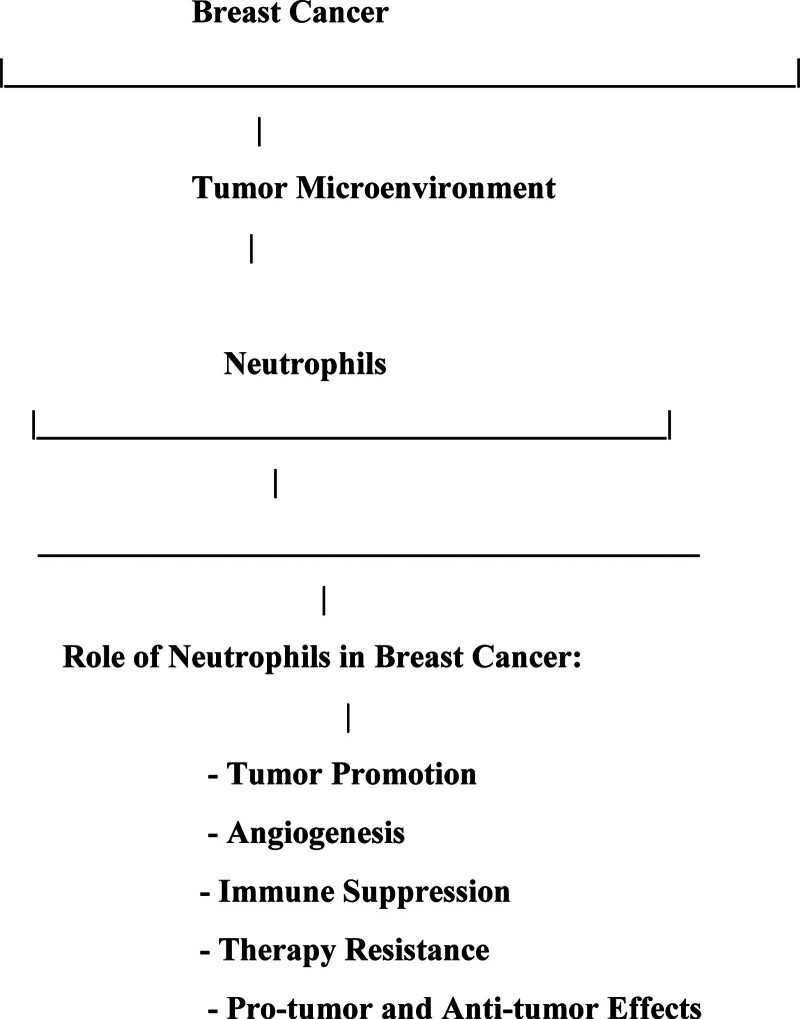
Central role of neutrophils within the tumor microenvironment of breast cancer.

Figure 1 represents the central role of neutrophils within the tumor microenvironment of breast cancer and highlights their diverse functions, including tumor promotion, angiogenesis, immune suppression, therapy resistance, and their ability to exert both pro-tumor and anti-tumor effects. Neutrophils’ involvement in various aspects of breast cancer underscores their significance in disease progression and treatment response.

## 9. Challenges and future directions of neutrophils in breast cancer

The study of neutrophils in breast cancer is a burgeoning field that holds promise for improving our understanding of tumor-immune interactions and developing novel therapeutic strategies. However, it also presents several challenges and unanswered questions. Neutrophils can exhibit diverse phenotypes and functions depending on the microenvironment.^[97]^ This heterogeneity makes it challenging to determine the overall impact of neutrophils on breast cancer and develop targeted therapies. Understanding the timing and staging of neutrophil involvement in breast cancer is critical.^[98]^ It is essential to determine when and how neutrophils influence tumor progression and whether their effects change as the disease evolves. Neutrophils’ role in breast cancer is highly context-dependent, influenced by factors like tumor subtype, genetic characteristics, and the immune landscape.^[99]^ Future research should aim to decipher these complexities.

Establishing reliable neutrophil-related biomarkers for breast cancer prognosis and therapeutic response prediction is an ongoing challenge. These markers need validation in large and diverse patient cohorts. Research is needed to develop immunotherapies that specifically target or modulate neutrophil functions in breast cancer. Identifying strategies to enhance the anti-tumorigenic activities of neutrophils while suppressing pro-tumorigenic functions is a key focus.^[61]^ Moving toward personalized treatment plans based on the specific neutrophil profile of each patient presents a challenge.^[100]^ Developing strategies to tailor therapies for individual patients is an important direction for future research. Exploring the potential for combination therapies that integrate neutrophil-targeting strategies with existing breast cancer treatments requires extensive investigation.^[101]^ These combinations need to be both safe and effective. A comprehensive understanding of the immune landscape in breast cancer, which includes the intricate interactions between different immune cells, is crucial. This understanding will help in optimizing immunotherapies and treatment strategies. Long-term, prospective studies that track changes in neutrophil profiles and functions over the course of breast cancer progression are needed. Such studies can provide insights into the dynamic nature of neutrophil-tumor interactions. Standardizing the methods and techniques used to study neutrophils in breast cancer is essential to ensure consistency and comparability across different research studies. Conducting clinical trials to evaluate the safety and efficacy of emerging neutrophil-targeted therapies in breast cancer is a crucial step for translating research findings into clinical practice.

Neutrophils in breast cancer is a promising area of research with clinical implications, but it comes with challenges related to heterogeneity, timing, context dependency, and the need for personalized treatments.^[102]^ Overcoming these challenges will require collaborative efforts from researchers, clinicians, and the pharmaceutical industry to advance our understanding of neutrophils in breast cancer and develop innovative therapeutic approaches.

## 10. Conclusion

The intricate role of neutrophils in breast cancer, as explored in this study, exemplifies the complex and dynamic nature of the tumor microenvironment and the immune response within this disease. Neutrophils, traditionally known as the foot soldiers of the innate immune system, have emerged as both allies and adversaries in the battle against breast cancer. Clinical implications have become increasingly evident, as neutrophil presence and behavior can serve as prognostic and predictive markers (highly predictive in ER+, HER2+ but not highly predictive in TNBCs). High levels of pro-tumorigenic neutrophil activities may be associated with poorer outcomes, suggesting the potential for targeted interventions. Immunotherapies and combination therapies aimed at modulating neutrophil functions hold promise in reshaping the landscape of breast cancer treatment.

The future directions in the study of neutrophils in breast cancer are poised to unlock even greater insights into the interactions between the immune system and the tumor. As research continues, we can anticipate more precise and effective therapeutic strategies that harness the full potential of these immune warriors. The ongoing quest to unveil the warriors within, the neutrophils, offers exciting prospects for improving breast cancer management, enhancing patient outcomes, and ultimately advancing the field of oncology.

## Author contributions

**Conceptualization:** Emmanuel Ifeanyi Obeagu.

**Methodology:** Emmanuel Ifeanyi Obeagu, Getrude Uzoma Obeagu.

**Supervision:** Emmanuel Ifeanyi Obeagu.

**Validation:** Emmanuel Ifeanyi Obeagu.

**Visualization:** Emmanuel Ifeanyi Obeagu.

**Writing – original draft:** Emmanuel Ifeanyi Obeagu, Getrude Uzoma Obeagu.

**Writing – review & editing:** Emmanuel Ifeanyi Obeagu, Getrude Uzoma Obeagu.
